# Performance Evaluation of Deep Learning-Based Prostate Cancer Screening Methods in Histopathological Images: Measuring the Impact of the Model’s Complexity on Its Processing Speed

**DOI:** 10.3390/s21041122

**Published:** 2021-02-05

**Authors:** Lourdes Duran-Lopez, Juan P. Dominguez-Morales, Antonio Rios-Navarro, Daniel Gutierrez-Galan, Angel Jimenez-Fernandez, Saturnino Vicente-Diaz, Alejandro Linares-Barranco

**Affiliations:** 1 Robotics and Tech. of Computers Lab, Universidad de Sevilla, 41012 Seville, Spain; jpdominguez@atc.us.es (J.P.D.-M.); arios@atc.us.es (A.R.-N.); dgutierrez@atc.us.es (D.G.-G.); ajimenez@atc.us.es (A.J.-F.); satur@us.es (S.V.-D.); alinares@atc.us.es (A.L.-B.); 2 Escuela Técnica Superior de Ingeniería Informática (ETSII), Universidad de Sevilla, 41012 Seville, Spain; 3 Escuela Politécnica Superior, Universidad de Sevilla, 41012 Seville, Spain; 4 Smart Computer Systems Research and Engineering Lab (SCORE), Research Institute of Computer Engineering (I3US), Universidad de Sevilla, 41012 Seville, Spain

**Keywords:** deep learning, convolutional neural networks, artificial intelligence, prostate cancer, performance evaluation, benchmark

## Abstract

Prostate cancer (PCa) is the second most frequently diagnosed cancer among men worldwide, with almost 1.3 million new cases and 360,000 deaths in 2018. As it has been estimated, its mortality will double by 2040, mostly in countries with limited resources. These numbers suggest that recent trends in deep learning-based computer-aided diagnosis could play an important role, serving as screening methods for PCa detection. These algorithms have already been used with histopathological images in many works, in which authors tend to focus on achieving high accuracy results for classifying between malignant and normal cases. These results are commonly obtained by training very deep and complex convolutional neural networks, which require high computing power and resources not only in this process, but also in the inference step. As the number of cases rises in regions with limited resources, reducing prediction time becomes more important. In this work, we measured the performance of current state-of-the-art models for PCa detection with a novel benchmark and compared the results with PROMETEO, a custom architecture that we proposed. The results of the comprehensive comparison show that using dedicated models for specific applications could be of great importance in the future.

## 1. Introduction

Prostate cancer (PCa) is the second most common cancer and the fifth leading cause of cancer death in men (GLOBOCAN [1]). In 2018, almost 1.3 million cases and around 360,000 deaths worldwide were registered due to this malignancy. According to the World Health Organization (WHO), there will be an increase of prostate cancer (PCa) cases worldwide, with 1,017,712 new cases being estimated for 2040. Most of these cases will be registered in Africa, Latin America, the Caribbean and Asia, and appear to be related to an increased life expectancy [2].

To diagnose PCa, digital rectal examination (DRE) is the primary test for the initial clinical assessment of the prostate. Then, prostate-specific antigen (PSA) is used in a screening method for the investigation of an abnormal prostatic nodule found in a digital rectal examination (DRE). Finally, in the case of abnormal DRE and elevated PSA results, trans-rectal ultrasound-guided biopsy is performed to obtain samples of the prostate tissue [3]. Then, these tissue samples are scanned, resulting on gigapixel-resolution images called whole-slide images (WSIs), which are then analyzed and diagnosed by pathologists.

Due to the high increment of new cases, and thanks to the impacts of Artificial Intelligence (AI) in recent years [4,5], several computer-aided diagnosis (CAD) systems have been developed to speed up the process of PCa diagnosis. A computer-aided diagnosis (CAD) system is an automatic or semi-automatic algorithm whose purpose is to assist doctors in the interpretation of medical images in order to provide a second opinion in the diagnosis. Among the different AI algorithms, deep learning (DL) has become very popular in recent years, and convolutional neural networks (CNNs) particularly [6]. They have been applied in several fields in medical image analysis, such as in disorder classification [7], lesion/tumor classification [8], disease recognition [9] and image construction/enhancement [10], among others.

Deep learning (DL) algorithms have also been applied to other medical image analysis fields such as histopathology, in which whole-slide images (WSIs) are used. Since it is not possible for a convolutional neural network (CNN) to work with a whole WSI as input due to its large size, a common approach is to divide this image into small subimages called patches. This procedure has been widely used in order to develop CAD systems in this field.

Recently, many researchers have investigated the application of CAD systems to the diagnosis of PCa in WSIs. Ström et al. [11] developed a deep learning (DL)-based CAD system to perform a binary classification distinguishing between malignant and normal tissue. The classification was performed using an ensemble of 30 widely used InceptionV3 models [12] pretrained on ImageNet. They achieved areas under the curve (AUC) of 0.997 and 0.986 on the validation and test subsets, respectively. For areas detected as malignant, the authors trained another ensemble of 30 InceptionV3 CNNs in order to discriminate between different PCa Gleason grading system (GGS) scores, achieving a mean pairwise kappa of 0.62 at slide level. Campanella et al. [13] presented a CAD system to detect malignant areas in WSIs. The classification was performed with the well-known ResNet34 model [14] together with a recurrent neural network (RNN) for tumor/normal classification. achieving an area under curve (AUC) of 0.986 at slide level. In a previous study [15], we proposed a CAD system, in which we focused on performing a patch-level classification of histopathological images between normal and malignant tissue. The proposed architecture, called PROMETEO, consisted of four convolution stages (convolution, batch normalization, activation and pooling layers) and three fully connected layers. The network achieved 99.98% accuracy, 99.98% F1 score and 0.999 AUC on a separate test set at patch level after training the network with a 3-fold cross-validation method.

These previous works achieved competitive results in terms of accuracy, precision and other commonly-used evaluation metrics. However, to the best of our knowledge, most state-of-the-art works do not focus on prioritizing the speed of the CAD system as an important factor. Many of them used very complex, well-known networks to train and test, without taking into account the computational cost and the time required to perform the whole process. Since these algorithms are not intended to replace pathologists but to assist them in their task, in some cases it is better to prioritize the speed of the analysis, sacrificing some precision so that the expert has a faster and more dynamic response from the system.

In this paper, a novel benchmark was designed in order to measure the processing and prediction time of a CNN architecture for a PCa screening task. First, the proposed benchmark was run for the PROMETEO architecture on different computing platforms in order to measure the impacts that their hardware components have on the WSI processing time. Then, using the personal computer (PC) configuration that achieved the best performance, the benchmark was run with different state-of-the-art CNN models, comparing them in terms of average prediction time both at patch level and at slide level, and also reporting the slowdown when compared to PROMETEO.

The rest of the paper is structured as follows: Section 2 introduces the materials and methods used in this work, including the dataset (Section 2.1), the CNN models (Section 2.2) and the benchmark proposed (Section 2.3). Then, the results obtained are presented in Section 3, which are divided in two different experiments: first, the performance of a proposed CNN model is evaluated in different platforms, and then it is compared to state-of-the-art, widely-known CNN architectures. Section 4 and Section 5 present the discussion and the conclusions of this work, respectively.

## 2. Materials and Methods

### 2.1. Dataset

In this work, a dataset with WSIs obtained from three different hospitals was used. These cases consisted of different Hematoxylin and Eosin-stained slides globally diagnosed as either normal or malignant.

From Virgen de Valme Hospital (Sevilla, Spain), 27 normal and 70 malignant cases obtained by means of needle core biopsy were digitized into WSIs. Clínic Barcelona Hospital (Barcelona, Spain) provided 100 normal and 129 malignant WSIs, also obtained by means of needle core biopsy. Finally, from Puerta del mar Hospital (Cádiz, Spain), 65 malignant (26 obtained from needle core biopsy and 39 from incisional biopsy) and 79 (33 obtained from needle core biopsy and 46 from incisional biopsy) WSIs were obtained. Table 1 summarizes the WSIs considered in the dataset.

### 2.2. CNN Models

Different CNNs models were considered in this work in order to compare their performance by using the benchmark proposed in Section 2.3. Three different architectures from state-of-the-art DL-based PCa detection works were compared, along with other well-known CNN architectures. The first one is the custom CNN model, called PROMETEO, which we proposed in [15], where we also demonstrated that applying stain-normalization algorithms to the patches in order to reduce color variability could improve the generalization of the model when predicting new unseen images from different hospitals and scanners. The second CNN architecture that was considered in this work is the well-known ResNet34 model [14], which was used by Campanella et al. in [13]. The third one is InceptionV3, introduced in [12], which was used by Ström et al. [11].

Apart from these three CNN models, other widely-known architectures were evaluated with the same benchmark, comparing their performance in terms of execution time with the rest of the networks for the same task. These were VGG16 and VGG19 [16], MobileNet [17], DenseNet121 [18], Xception [19] and ResNet101 [14].

### 2.3. Benchmark

In this work, a novel benchmark was designed in order to measure and compare the performances of different CNN models and platforms on a PCa screening task. In order to make the benchmark feasible to be shared with other researchers so that it could be run in different computers, a reduced set of WSIs were chosen from the dataset presented in Section 2.1. Since the total amount of WSIs of the dataset represent more than 300 gigabytes (GB) hard drive space, only 40 of them were considered, building up a benchmark of around 50 GB, which is much more shareable. These 40 WSIs were randomly selected, considering all the three different hospitals and scanners, and thus representing well the diversity of the dataset in this benchmark.

**Figure 1:**
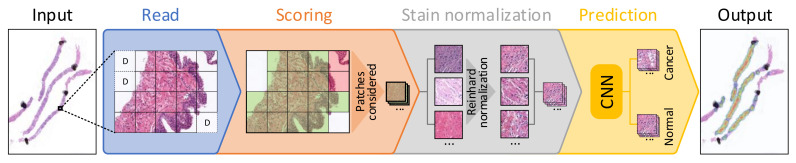
Block diagram detailing each of the steps considered for processing a whole-slide image (WSI) in the proposed benchmark.

The benchmark performs a set of processing steps which are detailed next (see Figure 1). First, as it was introduced in Section 1, since it is not possible for a CNN to use a whole WSI as input due to its large size, these images are divided into small subimages called patches (100 × 100 pixels at 10× magnification in this case), which are read from each WSI. This process is called "read," and apart from extracting the patches from the input WSI, those corresponding to background are discarded (identified as D in the figure). Then, in the scoring step, a score is given to each patch depending on three factors: the amount of tissue that it contains, the percentage of pixels that are within Hematoxylin and Eosin’s hue range and the dispersion of the saturation and brightness channels. This score allows discarding patches corresponding to unwanted areas, such as pen marks, external agents and patches with small amounts of tissue, among others. In Figure 1, discarded patches in this step are highlighted in red, while those that pass the scoring filter are highlighted in green. The third step, called stain normalization, performs a color normalization of the patch based on Reinhard’s stain-normalization algorithm [20,21] in order to reduce color variability between samples. In prediction, which is the last step of the process, each of the patches are used as input to a trained CNN, which classifies them as either malignant or normal tissue. Deeper insights into these steps are given in [15]. When the execution of the benchmark finishes, it reports both the hardware and system information of the computer used to run the benchmark, and the results of the execution. These results consist of the mean execution time and standard deviation for each of the four processes (read, scoring, stain normalization and prediction) shown in Figure 1 and presented in [15], both at patch level and at WSI level.

## 3. Results

The CNN-based PROMETEO architecture described in Section 2.2 was proposed and evaluated in terms of accuracy and many other evaluation metrics in [15]. In this work, we evaluated that model in terms of performance and execution time per patch and WSI.

First, the same architecture was tested in different platforms using the benchmark proposed in Section 2.3. These results allow us to measure and quantify the impacts of different components in the whole processing and prediction process, which is useful for designing an edge-computing prostate cancer detection system. Then, the benchmark was used to evaluate the performances of different state-of-the-art CNN architectures on the computing platform that achieved the best results on the first experiment.

**Figure 2:**
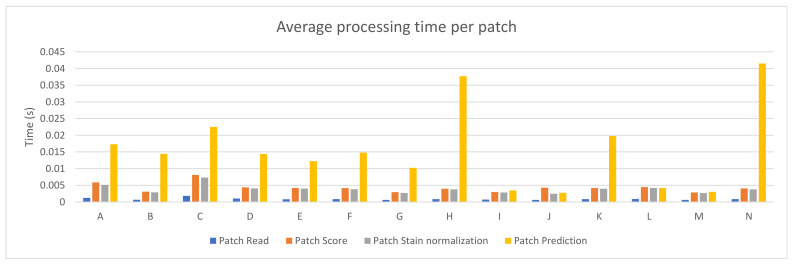
PROMETEO average patch processing time (in seconds) per step for each of the hardware configurations detailed in Table A1.

Fourteen different PC configurations were used to evaluate the performance of the PROMETEO architecture introduced in Section 2.2. The hardware specifications (central processing unit (CPU) and graphics processing unit (GPU)) of these computers are listed in Table A1 of Appendix A. In Figure 2, the average patch processing time is shown for each of the fourteen configurations, where the mean time for the steps performed when processing a patch (see Section 2.3) is reported. As it can be seen, the step that requires more time is the prediction in most of the cases, but it is highly reduced in configurations consisting of a GPU.

**Figure 3:**
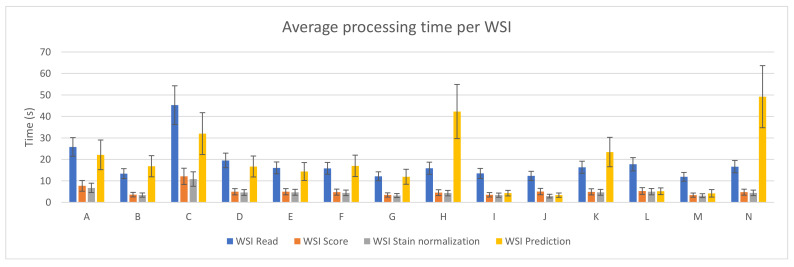
PROMETEO average WSI processing time (in seconds) and standard deviation per step for each of the hardware configurations detailed in Table A1.

Figure 3 depicts the average and standard deviation of the execution time needed per WSI when running the benchmark on the fourteen different PC configurations. As in Figure 2, each of the steps considered in the whole process is shown. As it can be seen, reading the whole WSI patch by patch is the step that involves the longest amount of time in most of the devices (mainly in those configurations with no GPU). This might seem contradictory considering Figure 2, but it is important to mention that, in that step, all patches from a WSI are read and analyzed, but not all of them are processed in the following steps. Unwanted areas, such as background regions with no tissue, are discarded before being scored. Then, only those which are not background and pass the scoring step are stain normalized and predicted by the CNN.

### 3.1. PROMETEO Evaluation

**Figure 4:**
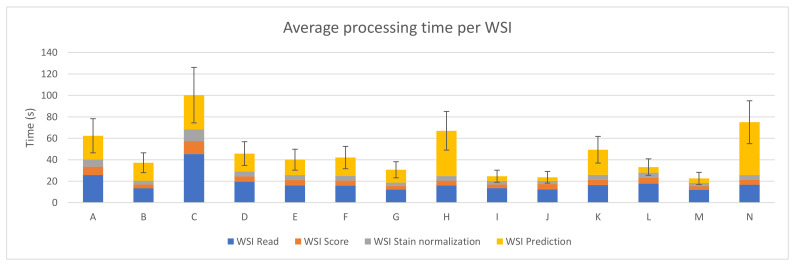
PROMETEO average WSI processing time (in seconds) and standard deviation of the hardware configurations detailed in Table A1.

The sum of the average execution time of the four preprocessing steps for each WSI was computed and it can be seen in Figure 4. The best case (device M) takes 22.56 ± 5.67 s on average to perform the whole process per WSI, where the prediction step only represents 4.20 ± 1.73 s.

The execution times obtained and used for generating the plots presented in this subsection are detailed in Table A2 of Appendix A.

### 3.2. Performance Comparison for Different State-of-the-Art Models

After evaluating the PROMETEO architecture using the benchmark designed for this work with different PCs, the same network was compared to other widely-known architectures. For this purpose, the same computer (device M) was used in order to perform a fair comparison. The same benchmark that was used in the previous evaluation (see Section 3.1) was executed in computer M (see Table A1) for each of the CNN architectures mentioned in Section 2.2. The CNNs considered are PROMETEO [15], ResNet34 and ResNet101 [14], InceptionV3 [12], VGG16 and VGG19 [16], MobileNet [17], DenseNet121 [18] and Xception [19].

**Figure 5:**
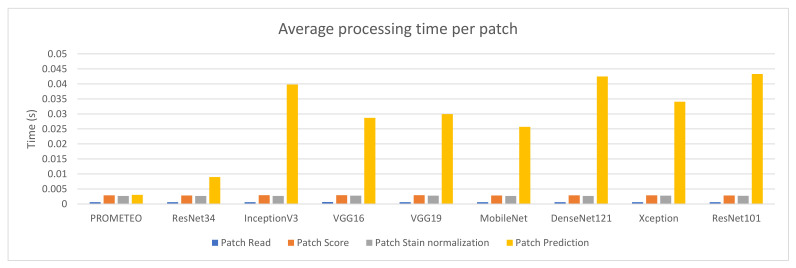
Average patch processing time (in seconds) per step for each of the CNN architectures using computer M (see Table A1).

The average patch processing time per preprocessing step can be seen in Figure 5 for each of the architectures mentioned. Since the architecture does not have an effect on the first three steps (reading the patch from the WSI, scoring it in order to discard unwanted patches, and normalizing it), the times needed to process them are similar across all the different cases reported in the figure. This does not happen with the prediction time, which directly depends on the complexity of the network.

**Figure 6:**
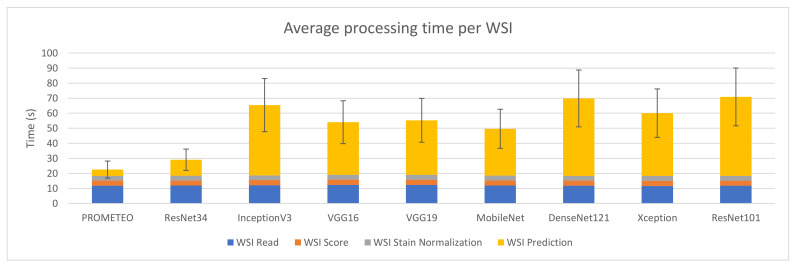
Average WSI processing time (in seconds) and standard deviation for each of the CNN architectures using computer M (see Table A1).

Figure 6 reports the combined processing time that device M takes to compute a WSI on average, together with its corresponding standard deviation. The same case explained in Section 3.1, where the WSI reading step takes much longer than the patch reading step in relation to the rest of the subprocesses, can also be observed in this figure. It is important to mention that the model proposed by the authors is faster than the rest in terms of prediction time, with a total of 22.56 ± 5.67 s per WSI on average.

Table 2 presents a summary of the results obtained for each architecture, focusing on the prediction process, which is the only one affected when changing the CNN architecture. Moreover, the number of trainable parameters and the slowdown are also reported. The latter is calculated by dividing the average prediction time per WSI of the corresponding CNN by that obtained with PROMETEO. This way, the improvement in terms of prediction time between PROMETEO and the rest of the architectures considered can be clearly seen. The proposed model predicts 2.55× faster than the CNN used in [13] and 11.68× faster than the one used in [11]. It is also important to mention that, in the latter, the authors did not use only an InceptionV3 model, but an ensemble of 30 of them. In this case, the figures and tables only report the execution times for a single network. When compared to other different widely-known architectures, PROMETEO is between 7.41× and 12.50× faster.

The execution times obtained and used for generating the plots presented in this subsection are detailed in Table A3 of Appendix B.

## 4. Discussion

**Figure 7:**
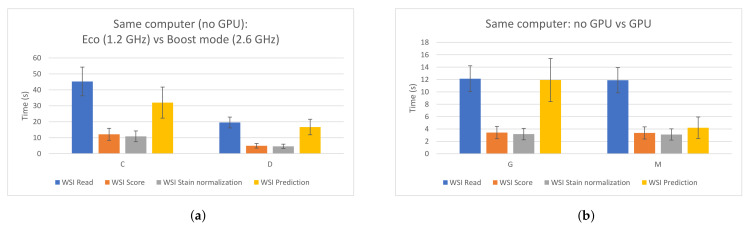
Impacts of the CPU and the GPU in the different WSI processing steps. (**a**) Same PC, different CPU frequency. Left: 1.2 GHz; right: 2.6 GHz. (**b**) Same PC. Left: without using GPU; right: using GPU.

In order to design a fast edge-computing platform for PCa detection, an evaluation of a proposed CNN was performed. This allowed us to compare different hardware components and configurations and measure the impacts of them when processing WSIs. Apart from the figures presented in Section 3.1, two specific cases are highlighted in Figure 7. Figure 7a shows the impact that the frequency of the CPU has on the whole process when using the same computer. As it can be seen, the four processing steps clearly benefit when a faster CPU is used. On the other hand, Figure 7b compares two cases where the same configuration is used, except for the GPU, which was removed in one of them. As expected, the GPU highly accelerated the prediction time (by around three times in this case). Therefore, in order to build a low-cost edge-computing platform for PCa diagnosis, this analysis could be useful and should be taken into account in order to prioritize in which component the funds should be invested. As it was explained, all patches from a WSI have to be read, but not all of them have to be predicted, since the majority of them correspond to background and are discarded first. Therefore, the CPU has a higher impact than the GPU in the whole process.

When comparing PROMETEO to other state-of-the-art CNN models, the former achieved the fastest prediction time, being from 2.55 times up to 12.50 times faster than any of the rest. Although the results in terms of accuracy and other commonly-used metrics in DL algorithms cannot be compared since the authors in [11,13,15] used different datasets, all of them reported state-of-the-art results for PCa detection. In [15], the authors compared PROMETEO to many of the models used in this work in terms of accuracy when using the same dataset for training and testing the CNN, showing that similar results were obtained.

The use of transfer learning in CNNs for medical image analysis has become a commonplace technique, and most of the current research focuses on using this approach for avoiding the problem of having to design, train and validate a custom CNN model for a specific task. This has proved to achieve state-of-the-art results in many different fields and has also accelerated the process of training a custom CNN from scratch [22]. However, when using this technique, very deep CNNs are commonly considered, which, as presented in this work, leads to a higher computational cost when predicting an input image, and therefore, a slower processing time. Some specific tasks could benefit from designing shallower custom CNN models from scratch, such as DL-based PCa screening, providing a faster response to the pathologists in order to help them in this laborious process. With the increases in the number of cases and the mortality produced by PCa, this factor could become even more relevant in the future.

As an alternative, cloud computing has provided powerful computational resources to big data processing and machine learning models [23]. Recent works have focused on accelerating CNN-based medical image processing tasks by using cloud solutions. While it is true that processing images using GPUs and tensor processing unit (TPUs) in the cloud is faster than in any local edge-computing device, there is an aspect that is not commonly taken into account when stating this fact: the time required to upload the image to the cloud. This depends on many factors and it is not easy to predict. Moreover, when digitizing histological images, scanners store them in a local hard drive using around 1 GB for each of them. As an example, with an upload speed of 300 Mbps, it would take more than 27 s in ideal conditions just for uploading the WSI to the cloud, which is more than the time it would take to fully process the image on a local platform.

To design a fast, low-cost, edge-computing platform, both the hardware components considered and the CNN model design have to be taken into account. Optimizing these two aspects led to achieving a very short WSI processing time when compared to current DL-based solutions without penalizing the performance of the system in terms of accuracy. In the next future, the authors would like to build a custom bare-bones approach based on the evaluations achieved in this work and test it in some of the hospitals that collaborated with us in this project.

## 5. Conclusions

In this work, we have presented a comprehensive evaluation of the performance of PROMETEO, a previously-proposed DL-based CNN architecture for PCa detection in histopathological images, which achieved 99.98% accuracy, 99.98% F1 score and 0.999 AUC on a separate test set at patch level.

Our proposed model outperforms other widely-used state-of-the-art CNN architectures such as ResNet34, InceptionV3, VGG16, VGG19, MobileNet, DenseNet121, Xception and ResNet101 in terms of prediction time. PROMETEO takes 22.56 s to predict a WSI on average, including the preprocessing steps needed, using an Intel^®^ Core™ i7-8700K (Intel, Santa Clara, CA, USA) and an NVIDIA^®^ GeForce™ GTX 1080 Ti (NVIDIA, Santa Clara, CA, USA). If we focus only on the prediction time, PROMETEO is between 2.55 and 12.50 times faster than any of the other architectures considered.

The promising results obtained suggest that edge-computing platforms and custom CNN designs could play important roles in the future for AI-based medical image analysis, being able to aid pathologists in their laborious tasks speed-wise.

## Figures and Tables

**Table 1:** Dataset summary.

Hospital	No. of WSIs		
	Normal	Malignant	Total
Virgen de Valme Hospital	27	70	97
Clínic Hospital	100	129	229
Puerta del Mar Hospital	79	65	144

**Table 2:** Average patch and WSI prediction time, slowdown and number of trainable parameters for each of the CNN architectures considered in this work.

Model	Avg. Prediction Time (patch)	Avg. Prediction Time (WSI)	Slowdown *	Trainable Parameters
PROMETEO	3.054 ± 4.845 ms	4.201 ± 1.739 s	1×	1,107,010
ResNet34	8.982 ± 10.086 ms	10.712 ± 3.134 s	2.55×	21,800,107
InceptionV3	41.301 ± 44.282 ms	49.076 ± 14.353 s	11.68×	23,851,784
VGG16	28.664 ± 9.241 ms	34.921 ± 10.160 s	8.31×	138,357,544
VGG19	29.931 ± 9.305 ms	36.250 ± 10.536 s	8.63×	143,667,240
MobileNet	25.689 ± 10.986 ms	31.110 ± 9.030 s	7.41×	4,253,864
DenseNet121	42.489 ± 16.859 ms	51.483 ± 14.945 s	12.25×	8,062,504
Xception	34.050 ± 11.789 ms	41.764 ± 12.175 s	9.94×	22,910,480
ResNet101	43.287 ± 14.679 ms	52.517 ± 15.266 s	12.50×	44,707,176

* Calculated by using the average prediction time per WSI and taking the PROMETEO architecture as reference. A slowdown of A× means that model B is A times slower than PROMETEO.

## Data Availability

Not applicable.

## Abbreviations

The following abbreviations are used in this manuscript:

AI	Artificial Intelligence
AUC	Area Under Curve
CAD	Computer-Aided Diagnosis
CNN	Convolutional Neural Network
CPU	Central Processing Unit
DL	Deep Learning
DRE	Digital Rectal Examination
GB	Gigabyte
GGS	Gleason Grading System
GPU	Graphic Processing Unit
H&E	Hematoxylin and Eosin
PC	Personal Computer
PCa	Prostate Cancer
PSA	Prostate-Specific Antigen
RNN	Recurrent Neural Network
TPU	Tensor Processing Unit
WHO	World Health Organization
WSI	Whole-Slide Image

## Appendix A. PROMETEO Evaluation

**Table A1:** Hardware specifications (CPU and GPU) of the different computers used in the PROMETEO evaluation.

Device	CPU	GPU
A	Intel^®^ Core™ i7-8850U @ 1.80 GHz 4 cores, 8 threads	-
B	Intel^®^ Core™ i9-7900X @ 3.30 GHz 10 cores, 20 threads	-
C	Intel^®^ Core™ i7-6700HQ @ 1.20 GHz 4 cores, 8 threads	-
D	Intel^®^ Core™ i7-6700HQ @ 2.60 GHz 4 cores, 8 threads	-
E	Intel^®^ Core™ i5-6500 @ 3.20 GHz 4 cores, 4 threads	-
F	Intel^®^ Core™ i7-4770K @ 3.50 GHz 4 cores, 8 threads	-
G	Intel^®^ Core™ i7-8700K @ 3.70 GHz 6 cores, 12 threads	-
H	Intel^®^ Core™ i7-4970 @ 3.60 GHz 4 cores, 8 threads	-
I	Intel^®^ Core™ i9-7900X @ 3.30 GHz 10 cores, 20 threads	NVIDIA^®^ GeForce™ GTX 1080 Ti 11 GB GDDR5X
J	AMD^®^ Ryzen™ 9 3900X @ 4.20 GHz 12 cores, 24 threads	NVIDIA^®^ GeForce™ GTX 1080 Ti 11 GB GDDR5X
K	Intel^®^ Core™ i5-6500 @ 3.20 GHz 4 cores, 4 threads	NVIDIA^®^ GeForce™ GT 730 2 GB GDDR5
L	Intel^®^ Core™ i7-4770K @ 3.50 GHz 4 cores, 8 threads	NVIDIA^®^ GeForce™ GTX 1080 Ti 11 GB GDDR5X
M	Intel^®^ Core™ i7-8700K @ 3.70 GHz 6 cores, 12 threads	NVIDIA^®^ GeForce™ GTX 1080 Ti 11 GB GDDR5X
N	Intel^®^ Core™ i7-4970 @ 3.60 GHz 4 cores, 8 threads	NVIDIA^®^ GeForce™ RTX 2060 6 GB GDDR6

**Table A2:** PROMETEO evaluation results. The average (Avg) and standard deviation (Std) of the execution times (in seconds) are shown for each of the four processes presented in Section 2.3 (Figure 1), both at patch level and at slide (WSI) level.

	Patch								WSI							
	Read		Score		Stain Normalization		Prediction		Read		Score		Stain Normalization		Prediction	
Device	Avg	Std	Avg	Std	Avg	Std	Avg	Std	Avg	Std	Avg	Std	Avg	Std	Avg	Std
A	0.00120757	0.00311363	0.00585298	0.00502059	0.00512905	0.00418173	0.01730045	0.00850617	25.8035576	4.33829335	7.68691321	2.50054828	6.74114185	2.1823579	22.1192591	6.90573801
B	0.00068973	0.00150109	0.00306787	0.0037015	0.00288733	0.00062438	0.01441587	0.00175146	13.3892811	2.29629633	3.59321811	1.04956324	3.38756321	0.98944353	16.8213335	4.91918301
C	0.00182337	0.00503892	0.00807697	0.00628436	0.00729532	0.00634226	0.02249318	0.0100416	45.2693313	8.99897452	12.1239614	3.77223369	10.8517522	3.36864663	31.9982855	9.74059672
D	0.00103901	0.00228084	0.00437608	0.00080403	0.00404258	0.00098854	0.01435914	0.00211298	19.5256697	3.39723828	4.94245166	1.44889791	4.59097219	1.34596044	16.6959336	4.87815493
E	0.00082695	0.00167953	0.00421429	0.00068222	0.00400594	0.00090984	0.01223286	0.00216942	16.0484505	2.76278471	4.94652829	1.4439207	4.70010431	1.37218657	14.3399619	4.1861481
F	0.00083281	0.00172759	0.00413688	0.00075105	0.0038354	0.00094666	0.01479934	0.00293383	15.8376234	2.74964356	4.7714866	1.3976735	4.42655776	1.29581397	16.9997911	4.98414625
G	0.00062777	0.00133961	0.00292148	0.00038463	0.00270451	0.00067159	0.0102172	0.00150291	12.1361434	2.0832663	3.42516114	1.00071198	3.17680098	0.92726679	11.9159923	3.48494303
H	0.00084291	0.00175864	0.00398322	0.00065638	0.0037566	0.00093803	0.03768491	0.00818776	15.8986914	2.81638821	4.5458395	1.34199731	4.29495389	1.26743003	42.2879135	12.595224
I	0.00069517	0.0015282	0.00299673	0.0003936	0.00285997	0.00066557	0.00345098	0.01023957	13.4663605	2.32710305	3.51854302	1.02719857	3.35297541	0.97965636	4.29145549	1.29811287
J	0.00062976	0.00137508	0.00428461	0.00027188	0.00246943	0.00060632	0.00275394	0.00906872	12.3392324	2.12166155	5.039479	1.47022453	2.91945068	0.85082711	3.35472003	1.00963885
K	0.00084153	0.00173514	0.00417708	0.00059019	0.00397734	0.00091936	0.01973523	0.01713999	16.462836	2.81694585	4.89897847	1.430121	4.67706547	1.36512005	23.4278429	6.84671918
L	0.00091354	0.00194007	0.00449805	0.00163909	0.00421455	0.00178876	0.0420623	0.01316229	17.743488	3.08314193	5.29275112	1.55927849	4.97026951	1.46232287	5.16441015	1.56004368
M	0.0006116	0.00129119	0.00286777	0.00039014	0.00265452	0.00068521	0.0030545	0.03559015	11.8833887	2.0393166	3.36354507	0.98195641	3.11514971	0.90982144	4.20128196	1.7395392
N	0.0008502	0.00498445	0.00405446	0.0007176	0.00375849	0.00097299	0.04150535	0.01602984	16.6332854	2.85486699	4.75020676	1.39576506	4.41236681	1.2946882	49.1934053	14.4530511

## Appendix B. Comparison between Different CNN Architectures

**Table A3:** Execution time comparison between different architectures. The average (Avg) and standard deviation (Std) of the execution times (in seconds) are shown for each of the four processes presented in Section 2.3 (Figure 1), both at patch level and at slide (WSI) level.

	Patch								WSI							
	Read		Score		Stain Normalization		Prediction		Read		Score		Stain Normalization		Prediction	
Architecture	Avg	Std	Avg	Std	Avg	Std	Avg	Std	Avg	Std	Avg	Std	Avg	Std	Avg	Std
PROMETEO	0.000612	0.001291	0.002868	0.00039	0.002655	0.000685	0.003054	0.004845	11.88339	2.039317	3.363545	0.981956	3.11515	0.909821	4.201282	1.739539
ResNet34	0.000612	0.001279	0.002844	0.000405	0.002676	0.000686	0.008982	0.010086	11.92095	2.045551	3.333316	0.973793	3.148634	0.918751	10.71205	3.134596
InceptionV3	0.000621	0.001317	0.002915	0.00041	0.002691	0.001136	0.039772	0.013828	12.10135	2.076446	3.415152	0.997178	3.168544	0.925316	46.72138	13.64997
VGG16	0.000635	0.001341	0.002931	0.000427	0.002785	0.000691	0.028664	0.009241	12.37371	2.140448	3.45475	1.007557	3.280768	0.957531	34.92197	10.16074
VGG19	0.000628	0.001313	0.002931	0.000425	0.00278	0.000682	0.029931	0.009305	12.34846	2.116793	3.44631	1.005834	3.266729	0.953449	36.25006	10.5361
MobileNet	0.000612	0.001278	0.00285	0.00042	0.002688	0.001111	0.025689	0.010986	11.96025	2.044115	3.383497	0.986745	3.208441	0.936362	31.11017	9.030854
DenseNet121	0.000611	0.001284	0.002879	0.000389	0.002687	0.000683	0.042489	0.01686	11.82392	2.035413	3.373127	0.985149	3.148681	0.919557	51.48291	14.94588
Xception	0.0006	0.001261	0.00288	0.000374	0.002758	0.000655	0.03405	0.011789	11.68313	1.987459	3.375536	0.986863	3.235289	0.945187	41.76486	12.17527
ResNet101	0.000607	0.001265	0.002839	0.000398	0.002701	0.00067	0.043287	0.014679	11.84637	2.035472	3.327598	0.971357	3.171104	0.925785	52.51713	15.26661
